# The Relationship between Eicosanoid Levels and Serum Levels of Metabolic and Hormonal Parameters Depending on the Presence of Metabolic Syndrome in Patients with Benign Prostatic Hyperplasia

**DOI:** 10.3390/ijerph16061006

**Published:** 2019-03-20

**Authors:** Katarzyna Grzesiak, Aleksandra Rył, Ewa Stachowska, Marcin Słojewski, Iwona Rotter, Weronika Ratajczak, Olimpia Sipak, Małgorzata Piasecka, Barbara Dołęgowska, Maria Laszczyńska

**Affiliations:** 1 Department of Histology and Developmental Biology, Pomeranian Medical University, Żołnierska 48, Szczecin 71-210, Poland; kasia.grzesiak302@gmail.com (K.G.); veronica.ratajczak@gmail.com (W.R.); mpiasecka@ipartner.com.pl (M.P.); maria@laszczynska.pl (M.L.); 2 Department of Medical Rehabilitation and Clinical Physiotherapy, Pomeranian Medical University, Żołnierska 54, Szczecin 71-210, Poland; iwrot@wp.pl; 3 Department of Biochemistry and Human Nutrition, Pomeranian Medical University, Broniewskiego 24, Szczecin 71-460, Poland; ewast@pum.edu.pl; 4 Department of Urology and Urological Oncology, Pomeranian Medical University, Powstańców Wlkp. 72, Szczecin 70-111, Poland; mslojewski@gmail.com; 5 Department of Obstetrics and Pathology of Pregnancy, Pomeranian Medical University, Żołnierska 48, Szczecin 71-210, Poland; olimpiasipak-szmigiel@wp.pl; 6 Department of Microbiology, Immunology, and Laboratory Medicine, Pomeranian Medical University, Powstańców Wlkp. 72, Szczecin 70-111, Poland; barbara.dolegowska@pum.edu.pl

**Keywords:** metabolic syndrome, benign prostatic hyperplasia, fatty acids

## Abstract

*Background*: The purpose of our investigation was to analyze the relationship between the serum levels of inflammatory mediators (HETE, HODE) and the levels of selected metabolic and hormonal parameters in patients with benign prostatic hyperplasia (BPH) with regard to concomitant metabolic syndrome (MetS). *Methods*: The study involved 151 men with BPH. Blood samples were taken for laboratory analysis of the serum levels of metabolic and hormonal parameters. Gas chromatography was performed using an Agilent Technologies 7890A GC System. *Results*: We found that waist circumference was the only parameter related to the levels of fatty acids, namely: 13(S)-HODE, 9(S)-HODE, 15(S)-HETE, 12(S)-HETE, and 5-HETE. In the patients with BPH and MetS, triglycerides correlated with 9(S)-HODE, 15(S)-HETE, 12(S)-HETE, and 5-HETE, which was not observed in the patients without MetS. Similarly, total cholesterol correlated with 9(S)-HODE, and 15(S)-HETE in the patients with BPH and MetS, but not in those without MetS. In the group of BPH patients with MetS, total testosterone positively correlated with 13(S)-HODE, and free testosterone with 9(S)-HODE. *Conclusions*: Based on this study, it can be concluded that lipid mediators of inflammation can influence the levels of biochemical and hormonal parameters, depending on the presence of MetS in BPH patients.

## 1. Background

Interest in metabolic syndrome (MetS) and inflammation as factors contributing to the risk of benign prostatic hyperplasia (BPH) has increased in recent years [1]. Numerous studies have confirmed the relationship between BPH and MetS [1,2,3]. Abnormalities occurring in the course of MetS lead to systemic inflammation and oxidative stress, and thus can be mediators between BPH and inflammation [1,4].

**Figure 1:**
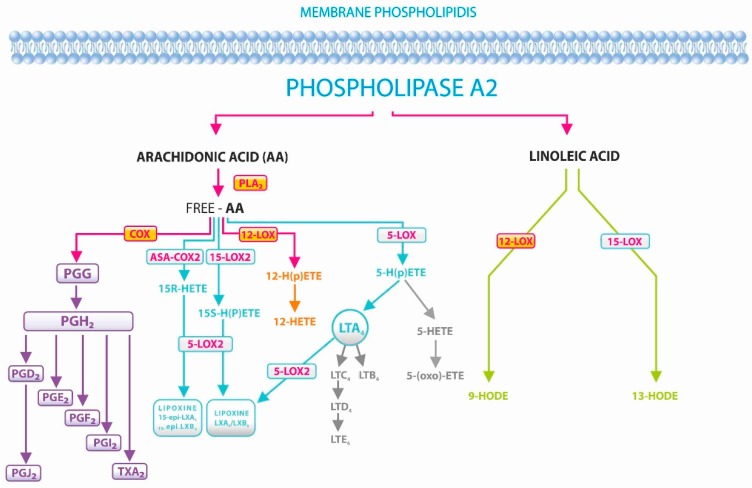
Metabolic pathways of arachidonic and linoleic acids in humans. Arachidonic and linoleic acid pathways. COX―cyclooxygenase; DiHETE―dihydroxyeicosatetraenoic acid; EET―epoxyeicosatrienoic acid; HETE―hydroxyeicosatetraenoic acid; HODE―hydroxyoctadecadienoic acid; LOX—lipoxygenase; LTX―lipoxin; PG―prostaglandin; TX―thromboxane.

The chief mediators of inflammation are arachidonic acid (AA) and linolenic acid (LA), as well as their oxidation products (Figure 1) [5,6], which include 5-oxo-eicosatetraenoic acid (5-oxo-ETE), 12- and 15-hydroxyeicosatetraenoic acids (12- and 15-HETE), and 9- and 13-hydroxy-octadecadienoic acids (9- and 13-HODE). 5-oxo-ETE is a strong eosinophilic chemoattractant and activator, synthesized not only in inflammatory cells but also in bronchial epithelial cells [7]. Its main effect is infiltration of eosinophils and―to a lower extend―neutrophils, which suggests that it plays a part in the pathophysiology of asthma and allergic diseases. The product of 12-lipoxygenase, 12(S)-HETE acts through the G protein-coupled receptor 31 (GPR31), and promotes proliferation and metastasis of cancer cells. Thus, it can be a target in cancer therapy. It can also act as a proinflammatory mediator in diabetes. On the contrary, 15(S)-HETE may protect against cancer [8]. Produced in granulocytes, 16-HETE has a vasodilatory effect [9], and is the first endogenous lipid inhibitor of adhesion and aggregation of neutrophils in humans [10]. 9- and 13-HODE have pleiotropic effects that can be either favorable or unfavorable, depending on the context [11]. Initially, 13-HODE increases lipid uptake, promotes reverse cholesterol transport, and induces apoptosis. The development of inflammation, associated with atherosclerosis, cancer, and MetS, is attributed to these acids [12]. They play a role in the signalization and regulation of inflammation in processes such as cell adhesion, chemotaxis and degranulation of neutrophils, macrophage superoxide production, activation of PPAR-c (peroxisome proliferator-activated receptor), and inhibition of protein kinase C [11,13,14,15]. Research on inflammatory mediators (HETE, HODE) in BPH patients with regard to MetS have not so far been described.

The purpose of our investigation was to analyze the relationship between the serum levels of inflammatory mediators (HETE, HODE) and the levels of selected metabolic and hormonal parameters in patients with BPH with regard to concomitant MetS.

## 2. Material and Methods

### 2.1. Patients

The study was carried out in a group of 151 patients with a diagnosis of BPH, aged 50–77 (mean ± SD: 66.99 ± 6.96). They were qualified, due to symptomatic BPH, for transurethral resection of the prostate (TURP) in the Clinic of Urology and Urologic Oncology, Pomeranian Medical University in Szczecin. Diagnosis was based on high International Prostate Score System questionnaire (IPSS), (long lasting symptoms, decreased Qmax in flow study or urinary retention. Men with cancer disease, active alcoholism, and thyroid diseases were excluded from the study. Neither Prostate Specific Antigen (PSA) level nor prostate volume routinely measured in every patient before admitting was taken into the consideration as contraindication for surgery, so we did not compare these parameters between study and control group. The study was approved by the Bioethical Commission of the Pomeranian Medical University in Szczecin (approval no. KB-0012/123/14). All patients gave informed written consent to take part in the study.

### 2.2. Clinical Examination

Anthropometric measurements—body weight, height, age, and waist circumference—were taken for all patients. The participants completed the questionnaire concerning their demographic data and chronic diseases. The men were divided into two groups: those with and those without MetS. MetS was diagnosed on the basis of the criteria proposed by the International Diabetes Federation (IDF) in 2005 [16]. The patients assigned to the MetS+ group had abdominal obesity ≥ 94 cm and at least two of the following abnormalities: triglycerides (TG) ≥ 150 mg/dL or treatment for dyslipidemia; HDL cholesterol < 40 mg/dL or treatment for dyslipidemia; fasting glycemia ≥ 100 mg/dL or treatment for type 2 diabetes; blood pressure ≥ 130/85 mmHg or treatment for hypertension. All components of MetS were considered both individually and as sets of symptoms.

Additionally, the body mass index (BMI) was calculated. Overweight was diagnosed if BMI ranged from 25 to 29.99 kg/m^2^, and obesity for BMI ≥ 30 kg/m^2^. The insulin resistance (HOMA-IR) index was gauged for nondiabetic patients according to the formula: fasting glucose (mmol/L) × fasting insulin (μU/mL)/22.5 [17].

### 2.3. Blood Serum Analysis

Nine mL blood samples were taken for laboratory analysis from a cubital vein on an empty stomach between 7:30 am and 9:00 am. Blood was collected using tubes with clot activator and gel separator. Next, it was centrifuged. A spectrophotometric method with commercial reagent kits (Biolabo, Aqua-Med, Łódź, Poland) was employed to determine the serum levels of fasting plasma glucose (FPG) for the nondiabetic men, as well as total cholesterol (TCh), low density lipoprotein (LDL) cholesterol, high-density lipoprotein (HDL) cholesterol, and triglycerides. The serum hormone levels—total testosterone (TT), free testosterone (FT), insulin (I), dehydroepiandrosterone sulphate (DHEAS), estradiol (E2), luteinizing hormone (LH), and sex hormone-binding globulin (SHBG)—were measured by the ELISA method using commercial reagent kits (DRG International, Marburg, Germany).

### 2.4. Isolation of Fatty Acids

Serum was obtained from blood clots centrifuged for 10 min at 1200 G. Fatty acids were extracted according to the Folch method [18]. Each 0.5 mL serum sample was saponified with 1 mL of 2 mol/L KOH methanolic solution (70 °C for 20 min), and methylated with 2 mL of 14% boron trifluoride in methanol under the same conditions. Next, 2 mL of *n*-hexane and 10 mL of saturated NaCl solution were added. 1 mL of the *n*-hexane phase was collected for analysis.

### 2.5. Analysis of Fatty Acid Methyl Esters

Gas chromatography was performed using an 7890A GC System (Agilent Technologies, Palo Alto, CA, USA) equipped with a SUPELCOWAX™ 10 Capillary GC Column (15 mm × 0.10 mm, 0.10 μm); Supelco, Bellefonte, PA, USA). Chromatographic conditions were as follows: the initial temperature was 60 °C for 0 min; it increased at a rate of 40 °C/min to 160 °C (0 min); next, it increased at a rate of 30 °C/min to 190 °C (0.5 min), and next at a rate of 30 °C/min to 230 °C (2.6 min). The entire analysis took about 8 min, and the gas flow rate was 0.8 mL/min with hydrogen used as a carrier gas. Identification of fatty acids was done by comparing their retention times with those of commercially available standards.

### 2.6. Statistical Analysis

Statistical analysis was performed using Statistica 12 software (StatSoft, Inc., Tulsa, OK, USA). Basic statistics (mean, standard deviation, minimum and maximum values) were applied to characterize the study sample. The Shapiro-Wilk test was employed to assess the normality of the distribution. Student’s *t*-test and the Mann-Whitney *U* test were applied to determine differences between the groups. Correlations between quantitative variables were calculated using Pearson’s correlation coefficient. The level of significance was set as *p* ≤ 0.05.

## 3. Results

We analyzed the relationship between MetS and lipid mediators of inflammation in BPH patients (Table 1). Our study demonstrated no statistically significant relationship between fatty acids and MetS as a whole syndrome in BPH patients.

The levels of fatty acids were also studied in terms of their relationship with abnormalities diagnosed in the course of MetS (Table 2). We found that abdominal circumference was the only parameter related to the levels of fatty acids, namely: 13(S)-HODE (*p* = 0.006), 9(S)-HODE (*p* = 0.004), 15(S)-HETE (*p* < 0.001), 12(S)-HETE (*p* = 0.039), and 5-HETE (*p* = 0.001).

Additionally, we analyzed correlations between anthropometric and metabolic parameters and the levels of the selected fatty acids in BPH patients with and without MetS (Table 3). In the BPH patients without MetS, 13(S)-HODE positively correlated with age. Body mass did not show significant correlations, while waist circumference positively correlated with 15(S)-HETE and 5-HETE in the BPH patients without MetS. In the patients with BPH and MetS, triglycerides correlated with 9(S)-HODE (*p* = 0.001), 15(S)-HETE (*p* = 0.001), 12(S)-HETE (*p* = 0.022), and 5-HETE (*p* = 0.001), which was not observed in the patients without MetS. Similarly, total cholesterol correlated with 9(S)-HODE (*p* = 0.001), and 15(S)-HETE (*p* = 0.003) in the patients with BPH and MetS, but not in those without MetS.

In the patients with MetS, the levels of fasting plasma glucose correlated with 9(S)-HODE (*p* = 0.001), 15(S)-HETE (*p* = 0.001), and 5-HETE (*p* = 0.001). In the patients without MetS this relationship was not observed. In the patients without MetS, waist circumference correlated with 15(S)-HETE (*p* = 0.025) and 5-HETE (*p* = 0.016), and fasting plasma glucose negatively correlated with 16RS-HETE (*p* = 0.01).

We also analyzed correlations between hormonal parameters and the levels of the selected fatty acids (Table 4). In the group of BPH patients with MetS, total testosterone positively correlated with 13(S)-HODE (*p* = 0.001), and free testosterone with 9(S)-HODE (*p* = 0.048). This relationship was not observed in the BPH patients without MetS.

## 4. Discussion

The literature review and the results of our study concerning lipid inflammatory markers and their impact on hormone levels in patients with BPH, depending on the presence of MetS, provide new information about the association between inflammation and MetS in BPH patients.

The link between fatty acids and MetS has thus far been described in relation to various diseases, including atherosclerosis [19], chronic renal disease [20], nonalcoholic fatty liver disease (NAFLD) [21], and systemic lupus [22]. Studies of the relationship between lipid inflammatory markers and prostate diseases have been based on comparative analysis of biochemical and metabolic parameters and the levels of fatty acids in BPH and prostate cancer [23,24,25]. Nevertheless, the association between fatty acids and MetS in BPH patients have not yet been elucidated.

Linolenic acid (LA) is converted by lipoxygenase (LOX) and cyclooxygenase (COX) into eicosanoids, which act as strong mediators of inflammation. This group of compounds includes lipoxins (LX), hydroxyeicosatetraenoic acids (HETEs), and hydroxyoctadecadienoic acids (HODEs) [26]. In our study, no statistically significant differences were demonstrated between the groups with reference to derivatives of fatty acids—eicosanoids: 16(R)/16(S)-HETE, 15(S)-HETE, 12(S)-HETE, 5(S)-oxo-ETE, 5(S)-HETE, 13(S)-HODE, 9(S)-HODE. MetS had no statistically significant influence on the levels of these acids in the patients with BPH. In our study, waist circumference was the only criterion for MetS that was statistically significantly related to acid levels. According to Martin et al. [27], 12- and 15-HETE levels are not statistically significantly related to visceral fat metabolism and fat deposition in the visceral region. In the study of Parsons et al. [28], visceral obesity positively correlated with prostate volume. Body weight, BMI, and waist circumference have been proved to positively correlate with prostate volume in many different populations [29,30,31].

Our investigation did not confirm a significant impact of type 2 diabetes on acid levels in the BPH patients. However, there is evidence that in the general population 12(S)-HETE can affect the activity of proinflammatory and proapoptotic cytokines, involved in the pathomechanism of diabetes [32].

Wang et al. [33] reported that patients with essential hypertension had elevated 13-HODE levels, which probably reflected increased oxidative stress. At the onset of atherosclerosis, 13-HODE is a dominant HODE, while at more advanced stages of the disease, the levels of 9-HODE are equally high or even higher. In our study, no other differences in acid levels were observed depending on hypertension. Morgantini et al. [34] informed that oxidized fatty acids, except for 12-HETE, negatively correlated with the ratio of HDL cholesterol to plasma apoA-I levels. This suggests that smaller molecules are enriched oxidized fatty acids.

Our correlation analysis showed that the levels of triglycerides were related to the levels of 9(S)-HODE, 15(S)-HETE, 12(S)-HETE, and 5-HETE in the patients with MetS. Additionally, in this group of patients, 9(S)-HODE and 15(S)-HETE positively correlated with fasting plasma glucose and cholesterol. Furthermore, fasting plasma glucose correlated with 5-HETE. Our results correspond with those obtained by other authors who studied atherogenic and diabetogenic effects of these acids [34,35]. In BPH patients without MetS, 13-HODE negatively correlate with triglycerides, and 16RS-HETE negatively correlate with fasting plasma glucose, which may prove their protective effect [36,37]. There is also a publication that confirms a positive effect of 20-HETE on insulin levels [38].

We found that eicosanoids were linked to hormonal parameters. 13(S)-HODE and 9(S)-HODE correlated with total testosterone and free testosterone respectively in the patients with MetS. The literature only provides publications concerning prostate cancer [39,40]. In the study of Ghosh et al. [41], 5-oxo-ETE inhibited apoptosis of prostate cancer cells, while 12-HETE stimulated neovascularization and proliferation of these cells [42]. The metabolism of linoleic acid by 15-lipoxygenase-1 (15-LOX-1), resulting in 13-HODE production, is higher in prostate cancer. The metabolism of 15-lipoxygenase-2 (15-LOX-2), and the level of its product, 15-HETE, on the other hand, are higher in normal prostate tissue. 15-LOX-2 and 15-HETE are supposed to have potential of suppressing carcinogenesis [43,44,45].

Adipose tissue is a place where triglycerides are accumulated, and hormones are secreted and synthesized. Additionally, it takes part in the metabolization of androgens. Adipose tissue enzymes are involved in the conversion of testosterone into its inactive metabolites. One of these enzymes is aromatase (ARO), whose activity has been demonstrated in adipose tissue, and which may also substantially contribute to visceral obesity, accumulation of fat, and MetS [46]. On the other hand, androgens influence the distribution and proliferation of adipose tissue. They also regulate the activity of lipoprotein lipase (LPL)—an enzyme involved in intracellular esterification of adipose tissue [47]. Present in adipose tissue, LPL hydrolyzes triglycerides circulating in the blood, which has an impact on the process of intracellular esterification and conversion of free fatty acids. The activity of LPL is decreased by androgens, which entails greater visceral obesity. Visceral adipose tissue (VAT) shows high expression of androgen receptors (AR). Obesity in men diagnosed on the basis of BMI correlates negatively with the level of testosterone and correlates positively with the level of estradiol. Aside from leptin, adipose tissue also contains other adipokines that may have an effect on Leydig’s cells synthesizing testosterone [46]. Currently, apart from playing the function of hormone metabolism and synthesis, adipose tissue is regarded as a new organ having immunomudulatory properties.

## 5. Conclusions

Based on this study, it can be concluded that lipid mediators of inflammation may influence the levels of biochemical and hormonal parameters, depending on the presence of MetS in BPH patients. It is worth emphasizing that MetS as a syndrome did not statistically significantly enhance the levels of acids in the patients with BPH, in the pathogenesis of which inflammation plays an important part. We did not analyze patients with MetS and without BPH. We also demonstrated the possibility of existing insignificant relationships between the levels of hormones and fatty acids, depending on MetS. The results of our investigation indicate the need for further research on the levels of fatty acids and their impact on the course and clinical picture of BPH with regard to MetS.

## Figures and Tables

**Table 1:** The relationship between MetS and lipid mediators of inflammation in BPH patients.

Variable	Patients with BPH and without MetS *n* = 99				Patients with BPH and with MetS *n* = 52				*p*
	X	SD	Min	Max	X	SD	Min	Max	
16RS HETE (µg/mL)	3.85	3.25	0.04	11.48	5.55	6.19	0.07	19.23	0.660
13S HODE (µg/mL)	0.20	0.34	0.02	2.40	0.29	0.70	0.01	4.88	0.226
9S HODE (µg/mL)	0.13	0.18	0.01	1.48	0.17	0.19	0.02	1.21	0.089
15S HETE (µg/mL)	0.98	1.08	0.10	8.44	0.99	0.91	0.10	5.76	0.485
12S HETE (µg/mL)	10.49	9.69	0.55	54.89	13.46	11.35	0.92	41.58	0.136
5 oxo ETE (µg/mL)	0.66	0.48	0.06	2.33	0.59	0.31	0.29	1.13	0.751
5 HETE (µg/mL)	0.15	0.12	0.02	0.66	0.18	0.15	0.02	0.81	0.186

MetS, metabolic syndrome; BPH; benign prostatic hyperplasia; X, arithmetic mean; SD, standard deviation; Min, mnimum; Max, maximum; *p*, statistical significance; *n*, number.

**Table 2:** The relationship between abnormalities diagnosed in the course of MetS and the levels of fatty acids.

**Variable**	**Abdominal Circumference below 94 cm** ***n* = 48**		**Abdominal Circumference above 94 cm** ***n* = 97**		***p***	**No Diabetes** ***n* = 112**		**Diabetes** ***n* = 39**		***p***
	**X**	**SD**	**X**	**SD**		**X**	**SD**	**X**	**SD**	
16RS HETE (µg/mL)	4.10	5.24	4.57	4.28	0.607	4.00	3.54	6.52	7.64	0.776
13S HODE (µg/mL)	0.16	0.33	0.26	0.56	0.006 *	0.19	0.32	0.35	0.83	0.242
9S HODE (µg/mL)	0.12	0.21	0.16	0.17	0.004 *	0.14	0.18	0.17	0.21	0.291
15S HETE (µg/mL)	0.72	0.78	1.11	1.10	<0.001 *	0.99	1.02	0.97	1.05	0.600
12S HETE (µg/mL)	9.09	8.65	12.73	11.01	0.039 *	10.92	9.21	13.49	13.46	0.703
5 oxo ETE (µg/mL)	0.56	0.15	0.66	0.47	0.908	0.65	0.47	0.60	0.33	0.973
5 HETE (µg/mL)	0.12	0.11	0.19	0.14	0.001 *	0.16	0.12	0.17	0.16	0.952
**Variable**	**No hypercholesterolemia** ***n* = 100**		**Hypercholesterolemia** ***n* = 51**		***p***	**No hypertension** ***n* = 51**		**Hypertension** ***n* = 100**		***p***
	**X**	**SD**	**X**	**SD**		**X**	**SD**	**X**	**SD**	
16RS HETE [µg/mL]	4.25	4.03	5.35	6.61	0.995	4.16	3.09	4.67	5.45	0.634
13S HODE [µg/mL]	0.22	0.37	0.26	0.71	0.377	0.18	0.32	0.25	0.57	0.501
9S HODE [µg/mL]	0.16	0.22	0.12	0.07	0.613	0.14	0.21	0.15	0.17	0.512
15S HETE [µg/mL]	1.04	1.18	0.87	0.53	0.739	1.00	0.86	0.97	1.11	0.364
12S HETE [µg/mL]	11.23	10.55	12.24	10.18	0.714	10.74	10.41	11.97	10.45	0.353
5 oxo ETE [µg/mL]	0.70	0.46	0.43	0.28	0.155	0.55	0.14	0.72	0.58	0.772
5 HETE [µg/mL]	0.17	0.14	0.16	0.12	0.838	0.16	0.13	0.16	0.14	0.731

X, arithmetic mean; SD, standard deviation; *p*, statistical significance; *, statistically significant parameter; *n*, number.

**Table 3:** Correlations between anthropometric and metabolic parameters and the levels of the selected fatty acids in BPH patients with and without MetS.

Variable		Correlations in Patients with BPH and MetS									Correlations in Patients with BPH and without MetS								
		Age	Body Weight	WC	TG	TCh	HDL	LDL	FPG	HOMA	Age	Body Weight	WC	TG	TCh	HDL	LDL	FPG	HOMA
16RS HETE	*P*	0.032	−0.049	−0.066	0.261	−0.088	−0.439	−0.075	−0.052	−0.007	−0.352	0.002	0.236	0.104	−0.258	−0.044	−0.256	−0.488 *	−0.166
	(*p*)	(0.913)	0.867)	(0.823)	(0.368)	(0.764)	(0.116)	(0.800)	(0.861)	(0.982)	(0.078)	(0.991)	(0.245)	(0.612)	(0.203)	(0.832)	(0.206)	(0.011)	(0.429)
13S HODE	*P*	−0.064	0.113	0.210	0.183	0.210	0.165	0.054	0.182	−0.052	0.213 *	−0.105	0.024	−0.208 *	0.117	0.174	0.106	0.045	0.004
	(*p*)	(0.657)	0.430)	(0.139)	(0.198)	(0.139)	(0.248)	(0.707)	(0.200)	(0.728)	(0.039)	(0.312)	(0.817)	(0.044)	(0.262)	(0.094)	(0.313)	(0.664)	(0.973)
9S HODE	*P*	0.098	0.005	0.114	0.672 *	0.480 *	−0.156	0.184	0.516 *	0.117	0.144	−0.026	0.080	−0.104	−0.069	0.054	−0.060	0.002	−0.041
	(*p*)	(0.488)	0.974)	(0.421)	(0.001)	(0.001)	(0.270)	(0.197)	(0.001)	(0.423)	(0.166)	(0.801)	(0.441)	(0.320)	(0.506)	(0.605)	(0.570)	(0.987)	(0.700)
15S HETE	*P*	0.079	−0.038	0.018	0.573 *	0.414 *	0.072	0.030	0.539 *	0.232	0.140	0.031	0.232 *	−0.100	0.022	0.081	0.025	−0.023	−0.010
	(*p*)	(0.583)	0.792)	(0.899)	(0.001)	(0.003)	(0.614)	(0.834)	(0.001)	(0.112)	(0.181)	(0.771)	(0.025)	(0.339)	(0.837)	(0.440)	(0.810)	(0.826)	(0.923)
12S HETE	*P*	0.214	−0.097	−0.070	0.318 *	0.113	0.011	−0.100	0.099	−0.128	0.149	0.007	0.111	−0.008	−0.097	−0.151	−0.039	−0.105	−0.112
	(*p*)	(0.127)	0.495)	(0.621)	(0.022)	(0.423)	(0.939)	(0.486)	(0.486)	(0.381)	(0.153)	(0.945)	(0.291)	(0.937)	(0.357)	(0.149)	(0.712)	(0.317)	(0.291)
5 HETE	*P*	0.180	0.029	0.135	0.543 *	0.272	−0.154	−0.038	0.336 *	0.110	0.182	0.077	0.251 *	−0.031	−0.005	−0.030	0.022	0.011	−0.021
	(*p*)	(0.205)	0.840)	(0.346)	(0.001)	(0.054)	(0.279)	(0.791)	(0.016)	(0.457)	(0.083)	(0.466)	(0.016)	(0.769)	(0.966)	(0.779)	(0.833)	(0.921)	(0.845)

*P*, correlation coefficient; *p*, statistical significance; MetS, metabolic syndrome; BPH; benign prostatic hyperplasia; WC, waist circumference; TG, triglyceride; TCh, total cholesterol; LDL, low-density lipoprotein; HDL, high-density lipoprotein; FPG, fasting plasma glucose; HOMA, insulin resistance; *, statistically significant parameter; *n*, number.

**Table 4:** Correlations between hormonal parameters and the levels of the selected polyunsaturated fatty acids in BPH patients with and without MetS.

Variable		Correlations in Patients with BPH and MetS								Correlations in Patients with BPH and without MetS							
		DHEAS	E2	SHBG	LH	TT	TF	IGF1	I	DHEAS	E2	SHBG	LH	TT	TF	IGF1	I
16RS HETE	*P*	−0.074	0.163	−0.117	0.480	−0.202	−0.029	0.140	−0.236	0.105	0.025	0.045	−0.125	−0.054	−0.021	−0.130	−0.048
	(*p*)	(0.801)	(0.577)	(0.691)	(0.082)	(0.489)	(0.921)	(0.632)	(0.437)	(0.610)	(0.904)	(0.827)	(0.541)	(0.793)	(0.919)	(0.528)	(0.827)
13S HODE	*P*	0.028	0.046	0.135	0.002	0.447 *	0.259	0.164	−0.167	−0.013	−0.058	0.073	0.088	−0.114	−0.092	0.117	−0.019
	(*p*)	(0.846)	(0.751)	(0.346)	(0.990)	(0.001)	(0.066)	(0.250)	(0.302)	(0.899)	(0.578)	(0.486)	(0.401)	(0.275)	(0.379)	(0.263)	(0.865)
9S HODE	*P*	0.050	−0.061	0.072	0.126	0.059	0.275 *	0.059	−0.053	−0.009	−0.062	0.104	−0.054	−0.035	−0.046	0.093	−0.061
	(*p*)	(0.724)	(0.668)	(0.614)	(0.372)	(0.677)	(0.048)	(0.677)	(0.743)	(0.935)	(0.550)	(0.317)	(0.607)	(0.739)	(0.662)	(0.373)	(0.594)
15S HETE	*P*	0.007	−0.095	−0.102	0.020	−0.054	0.253	−0.037	0.046	−0.024	−0.061	0.097	−0.082	0.004	−0.049	0.070	−0.048
	(*p*)	(0.962)	(0.505)	(0.478)	(0.890)	(0.709)	(0.074)	(0.796)	(0.776)	(0.822)	(0.561)	(0.356)	(0.433)	(0.966)	(0.643)	(0.508)	(0.676)
12S HETE	*P*	−0.161	−0.110	0.068	0.139	0.099	0.060	0.116	−0.239	−0.033	0.004	0.121	−0.164	0.069	−0.058	−0.022	−0.124
	(*p*)	(0.253)	(0.439)	(0.632)	(0.324)	(0.487)	(0.672)	(0.414)	(0.132)	(0.753)	(0.972)	(0.248)	(0.117)	(0.508)	(0.579)	(0.833)	(0.279)
5 HETE	*P*	−0.107	−0.076	−0.034	0.120	0.016	0.190	0.167	−0.054	0.043	−0.013	0.141	−0.076	0.129	−0.012	0.075	−0.045
	(*p*)	(0.454)	(0.597)	(0.815)	(0.404)	(0.910)	(0.181)	(0.242)	(0.740)	(0.685)	(0.905)	(0.180)	(0.470)	(0.220)	(0.907)	(0.476)	(0.700)

*P*, correlation coefficient; *p*, statistical significance; MetS, metabolic syndrome; BPH; benign prostatic hyperplasia; TT, total testosterone; TF, free testosterone; SHBG, sex hormone binding globulin; E2, estradiol; DHEAs, dehydroepiandrosterone sulfate; LH, luteinizing hormone; IGI-1, insulin like grow factor-1; I, insulin; *, statistically significant parameter; *n*, number.
